# Low cigarette smoking prevalence in peri-urban Peru: results from a population-based study of tobacco use by self-report and urine cotinine

**DOI:** 10.1186/s12971-017-0137-8

**Published:** 2017-07-21

**Authors:** Brooks W. Morgan, Kathryn M. Leifheit, Karina M. Romero, Robert H. Gilman, Antonio Bernabe-Ortiz, J. Jaime Miranda, Harold I. Feldman, John J. Lima, William Checkley, CRONICAS Cohort Study

**Affiliations:** 1 Division of Pulmonary and Critical Care, School of Medicine, Johns Hopkins University, 1830 E. Monument St, Baltimore, Maryland 21205 USA; 2 Department of Epidemiology, Bloomberg School of Public Health, Johns Hopkins University, 615 N. Wolfe St, Baltimore, Maryland 21205 USA; 3 Biomedical Research Unit, A.B. PRISMA, Carlos Gonzales 251, 15088 San Miguel, Peru; 4 Program in Global Disease Epidemiology and Control, Department of International Health, Bloomberg School of Public Health, Johns Hopkins University, 615 N. Wolfe St, Baltimore, Maryland 21205 USA; 5 CRONICAS Center of Excellence in Chronic Diseases, Universidad Peruana Cayetano Heredia, 31 Av. Honorio Delgado 430, 15102 Lima, Peru; 6 Department of Biostatistics and Epidemiology, Perelman School of Medicine at the University of Pennsylvania, 3400 Civic Center Blvd, Philadelphia, Pennsylvania 19104 USA; 7 Center for Pharmacogenomics and Translational Research, Nemours Children Clinic, 14785 Old St. Augustine Rd, Jacksonville, Florida 32258 USA

**Keywords:** Tobacco, Smoking, Cotinine, Epidemiology

## Abstract

**Background:**

A recent study found lower self-reported prevalence of tobacco smoking in a peri-urban area of Lima, Peru than previously reported in urban samples. These regions encompass substantial proportions of Peru’s population – ones at greater risk of disease due to reduced healthcare access – but have been less often studied. We validate low smoking prevalence with urine cotinine and characterize chronic disease and lung function outcomes between non-, occasional, and daily smokers.

**Methods:**

Data are from the CRONICAS Cohort Study, a population-based longitudinal study in four low-resource Peruvian settings, which began in 2010. Of a baseline cohort of 2978 adults, we prospectively followed 2583 (87%) to determine prevalence of chronic illness.

**Results:**

In a baseline sub-sample of 382 participants, median adjusted cotinine was 0.0 mcg/mg (IQR 0–0) for both self-reported non-smokers and occasional smokers compared to 172.3 mcg/mg (IQR 0–709.2) for daily smokers. Creatinine-adjusted cotinine validated daily smoking prevalence of 4.7% at a cutoff of 100 mcg/mg. Kappa statistic for daily smoking and creatinine- adjusted cotinine ≥100 mcg/mg was 0.65 (95% CI 0.47, 0.83), indicating substantial agreement. At baseline, we found 3.3% daily and 8.9% occasional smoking by self-report for the full cohort. Follow-up indicated little difference in chronic disease prevalence between groups. Daily smokers trended toward having a greater decline in FVC (−1%; 95% CI -2.9, 0.8) and FEV_1_ (−1.3%; 95% CI -3.2, 0.6) over 40 months when compared to non-smokers, whereas the decline in lung function for occasional smokers was similar compared to non-smokers (−0.2% FVC; 95% CI -1.5, 1.0) and (0% FEV_1_; 95% CI -1.3, 1.3).

**Conclusions:**

Our data places Peru within a previously-described pattern of smoking found in much of Latin America, favoring occasional over daily smoking and low cigarette consumption. We determine that there are not significant differences between smoking groups concerning chronic disease outcomes. We favor distinguishing between daily and occasional smokers in order to accurately characterize these low-use populations.

**Electronic supplementary material:**

The online version of this article (doi:10.1186/s12971-017-0137-8) contains supplementary material, which is available to authorized users.

## Background

While tobacco smoking has declined dramatically in high-income countries, data suggests that it may be increasing in low- and middle-income countries (LMICs). This rise in smoking stands to accelerate countries’ epidemiologic transitions, contributing to increasing rates of chronic disease morbidity and mortality [1, 2]. In these countries, accurate tobacco use data is essential for public health practitioners designing and implementing interventions to curb smoking and prevent chronic disease.

This work focuses on Peru as a case study. Peru is an LMIC showing early signs of increasing chronic disease, but an uncertain smoking landscape. Earlier studies that have attempted to estimate the prevalence of smoking in Peru have largely focused on urban areas, overlooking peri-urban shantytowns. The CARMELA study reported that the prevalence of current tobacco use – roughly, anyone who reported current daily or occasional inhaled tobacco use – among those aged 25–64 years in urban Lima, Peru was 26.6% in 2005 [3]. The same year, the Center for Information and Education for the Prevention of Drug Abuse in Peru reported a 27.7% prevalence of all smoking and a 7.7% prevalence of daily smoking among those aged 12–64 years, also in urban Lima [4]. Meanwhile, our group estimated in a 2012 study that smoking prevalence may be substantially lower in peri-urban Lima, with a prevalence estimate of 16% for cigarette smoking among adults ≥40 years old [5]. Importantly, only 2% of the sample were daily smokers with the balance reporting occasional smoking [5]. This finding, if true, has important public health implications for Peru. Given the increasing proportion of Peruvians residing in peri-urban zones, high-quality surveillance must be directed to these areas to derive estimates of chronic disease prevalence and risk.

Our first objective was to describe the tobacco-using population of peri-urban Peru by validating our low self-reported finding smoking prevalence with urine cotinine. Cotinine, the primary metabolite of nicotine, is a reliable [6] and valid [7, 8] biomarker that has been long used as a surrogate for tobacco smoke exposure in the previous 48 h [9–22]. We compared the prevalence of smoking by self-report with that based on a laboratory analysis of urine cotinine in a population-based sample of 382 adults from low-income peri-urban Lima and semi-urban Tumbes, Peru. Our second objective was to characterize the chronic disease burden in Peru, especially related to smoking status. To accomplish this, we compared chronic disease diagnoses, respiratory symptoms, biomarkers, and lung function scores over a 40-month follow up period between self-reported daily, occasional, and non-smokers over four separate field sites. For this analysis, we utilized our full cohort of 2978 adult participants.

## Methods

### Study design and setting

We utilized self-report and biomarker data collected at baseline in a random subsample of participants in the CRONICAS cohort, a prospective longitudinal study on cardiovascular disease, hypertension, diabetes, and lung function for which data collection began in September 2010 [23]. Recruitment sites were chosen to represent a cross-section of low-income populations in Peru. These areas represent a large percentage of Peru’s population and have access to fewer health resources; however, they are less often studied as they are not as accessible as urban populations [5]. Pampas de San Juan de Miraflores is a physically diverse, peri-urban community 25 km south of Lima’s city center, and home to a large population of Andean immigrants which, studies have shown, suffer worsening cardiovascular risk profiles with urbanization similar to lifelong urban residents [24]. It represents a somewhat urbanized location at sea level with high ambient air pollution and low use of biomass fuels. Tumbes, on Peru’s northern coast, is a mix of rural areas and growing urban sections. It is much less densely populated than the Lima site and represents a semi-urban location at sea level with low ambient air pollution and high biomass fuel use. Puno, in southeastern Peru, is home to two recruitment sites of 500 participants each, one rural and one urban. They are both at high altitude and feature low ambient air pollution; however, biomass fuel use, while rare in the urban site, is prevalent in the rural site.

### Participants, recruitment, and ethics

Individuals were randomly selected from census data to form simple age- and sex-stratified cohorts of around 1000 participants per site. Study personnel visited the households of selected individuals to invite them to participate, ascertain oral consent (due to high rates of illiteracy), and perform baseline data collection. A random sub-sample of participants from Lima and Tumbes was invited to participate in ancillary studies (Puno was not included due to financial constraints). Inclusion criteria included age ≥ 35 years and permanent residency of the area. Exclusion criteria included pregnancy, physical or mental disability (sufficient to impede implementation of the study protocol), and active pulmonary tuberculosis. The study protocol was reviewed and approved by the Institutional Review Boards of Johns Hopkins University in Baltimore, USA, and A.B. PRISMA and Universidad Peruana Cayetano Heredia in Lima, Peru.

### Data collection

Data collection is described in detail elsewhere [23]. Briefly, baseline data collected during the recruitment visit included demographic, socioeconomic, and lifestyle risk factors (including smoking status); biomass exposure; and cardiovascular and respiratory symptoms, and was modified from WHO’s STEP questionnaire for non-communicable disease and the Global Adult Tobacco Survey (GATS) [25, 26]. During the initial visit, appointments were made for a clinical evaluation. At the clinic, lung function was ascertained with a portable spirometer (Easy-On-PC, ndd, Zurich, Switzerland) both pre- and post-administration of salbutamol. Blood and urine were collected by a trained technician. The date of collection of biological samples ranged from 22 days before survey to 228 days afterward. Whole blood and plasma were collected in ethylenediaminetetraacetic acid (EDTA) containing tubes and sodium fluoride/EDTA tubes, respectively, while urine was collected in 15 mL containers. Samples were stored at 4–8 °C for two weeks before being moved to a storage facility where the urine was separated into four 1.5 mL vials. Samples were then kept at −20 °C until laboratory analysis. Our biological samples were all analyzed in a single laboratory. Assay quality was validated against external standards and internal duplicate assays and were monitored by BioRad (http://www.bio-rad.com). Methods for the measurement of plasma glucose, serum insulin, hemoglobin A1c, total cholesterol, and HDL cholesterol are outlined in the parent sub-study protocol [27]. Urine creatinine was measured via modified kinetic Jaffé method. Urine cotinine was analyzed via gas chromatography-mass spectrometry [28]. The limit for detection was 0.16 ng/mL. We present urine cotinine both unadjusted and standardized by level of urine creatinine (cotinine/creatinine ratio).

### Definitions

Self-reported smoking was defined by the answer to the question, “Do you currently smoke cigarettes daily, occasionally, or not at all?” Current smoking was defined as a response of “Occasionally” (<1 cigarette/day) or “Daily” (1+ cigarettes/day) while daily (active) smoking was defined as only those who responded “Daily.” Based on previous research, adjusted urine cotinine was categorized into “none” (<10 mcg/mg), “low” (10–100 mcg/mg), and “high” (≥100 mcg/mg), which were used as references for our classifications of non-smoking, occasional/passive smoking, and daily (active) smoking, respectively [20, 29–33].

Fasting glucose, insulin, HbA1c, HDL, LDL, total cholesterol, and triglycerides were taken from blood samples. Diabetes mellitus was defined as self-reported physician diagnosis, use of anti-diabetic medications, or fasting plasma glucose ≥126 mg/dL [34]. Hypertension was defined as self-reported physician diagnosis, systolic blood pressure (SBP) ≥140 mmHg, diastolic blood pressure (DBP) ≥90 mmHg, or receipt of anti-hypertensive therapy [35].

Insulin resistance was assessed using the homeostasis model assessment (HOMA-IR) by Matthews et al. [36]. The Framingham risk score (FRS) was calculated from the National Cholesterol Education Program Adult Treatment Panel III algorithm, which is based on cardiovascular risk factors, including age, sex, total cholesterol, HDL-cholesterol, systolic BP and smoking status [37]. Socioeconomic status was assessed using a wealth index based upon occupation, assets, and household income and facilities [38]. Body mass index (BMI) was calculated as weight (kg) divided by height (m) squared.

### Biostatistical methods

To validate the prevalence of active smokers in peri-urban areas, prevalence estimates are reported as means with 95% confidence intervals. Differences between recruitment sites were examined using Chi-square tests for categorical variables and Wilcoxon rank-sum tests or Student’s t-tests for continuous variables. Agreement between self-reported smoking and adjusted cotinine categories were assessed with Fleiss’ kappa statistic [39]. To characterize smoking populations, multivariable regressions were performed to assess the relationship between self-reported smoking status at baseline and health outcomes over follow-up. Continuous variables were analyzed with generalized linear mixed effects longitudinal models for change in means while logistic random effects models were used for binary variables. For all outcomes, the relevant chronic disease diagnosis, symptom, or biomarker was modeled as the dependent variable with smoking status as the independent predictor. To isolate the effects of smoking status, models included age, sex, site, and wealth index as covariates. Only those with complete follow-up were included in analyses. All analyses were conducted in STATA version 13 (StataCorp, College Station, Texas, USA).

## Results

### Participant characteristics

A total of 404 agreed to participate in the subsample, of which 50.2% were male with a mean age of 54.9 years old. Twenty-two were excluded from analysis for missing urine cotinine values. Overall, there were 2978 adult participants in the CRONICAS cohort with complete baseline data for analysis and 382 in a baseline subset of participants with urine cotinine values. Neither sex nor age differed between sites, though smoking did (Table 1). The cotinine sub-sample did not significantly vary from the balance of the parent sample (Lima and Tumbes) in any measure (Additional file 1: Table S1).

**Table 1:** Demographic characteristics of recruitment sites and *p*-value for difference between sites, CRONICAS Study, Peru, 2010

	Lima	Tumbes	Urban Puno	Rural Puno	*p*-value
*n* (%)	1004 (33.7)	966 (32.4)	503 (16.9)	506 (17.0)	
Age, median (IQR)	54.7 (45.4–64.1)	54.7 (44.7–64.6)	54.8 (44.9–64.4)	54.8 (45.1–63.9)	0.82
Male, *n* (%)	492 (49.0)	483 (50.0)	249 (49.5)	239 (47.4)	0.82
Daily use of biomass fuels, *n* (%)	60 (6.0)	221 (23.4)	25 (4.8)	483 (96.6)	**<0.001**
Education in years, *n* (%)					**<0.001**
Primary or Less	424 (42.2)	524 (54.3)	72 (14.3)	318 (62.9)	
Secondary	403 (40.1)	296 (30.7)	139 (27.6)	158 (31.2)	
Higher than Secondary	177 (17.6)	145 (15.0)	292 (58.1)	30 (5.9)	
Wealth Index, *n* (%)					**<0.001**
Low	121 (12.1)	311 (32.3)	120 (23.9)	356 (70.4)	
Medium	366 (36.5)	393 (40.9)	129 (25.7)	136 (26.9)	
High	517 (51.5)	258 (26.8)	254 (50.5)	14 (2.8)	
Smoking, *n* (%)					**<0.001**
Non-smoking	855 (85.2)	843 (87.4)	447 (88.9)	469 (92.7)	
Occasional	116 (11.6)	68 (7.1)	45 (9.0)	6 (7.1)	
Daily	33 (3.3)	54 (5.6)	11 (2.2)	1 (0.2)	
Pack-years (ever smokers), median (IQR)	0.1 (0.0–0.8)	0.5 (0.1–4.6)	0.1 (0.0–1.2)	0.1 (0.0–0.3)	**<0.001**

Bold indicates significance at *p* = 0.05. Differences estimated by t-tests, Wilcoxon rank-sum, or Chi-squared tests

A total of 2583 participants (86.7%) remained in the study through 40-month follow up. The balance of 395 (13.3%) was lost to follow up, twenty-three of whom were deceased. Most notably, participants from urban and rural Puno were more likely to be lost to follow up than those from Lima or Tumbes (Additional file 1: Table S2).

Among our full cohort at baseline, prevalence of current smoking was about 16.5% for those aged 35–54 years. This fell to 10% among those aged 55–64 years and 5.5% for those aged ≥65 years (Additional file 1: Table S3). Men were three to seven times more likely to be current or daily smokers than women, with variations by age and site. Daily smokers comprised 99 of the 364 self-reported current smokers (27.2%). Occasional smokers consumed a mean of 0.8 cigarettes per day while daily smokers consumed a mean of 4.3 cigarettes per day. Among occasional smokers, men consumed more cigarettes, but among daily smokers, males and females consumed the same. The highest mean number of cigarettes smoked per day by all current smokers was in Tumbes (2.2), followed by urban Puno (1.8), Lima (1.7), and rural Puno (0.2).

### Cotinine and self-reported smoking

**Fig. 1.**
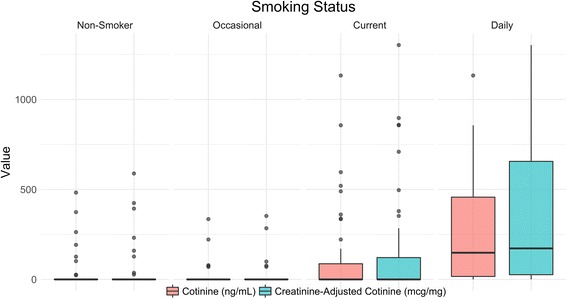
Comparison of urine cotinine and adjusted cotinine values between smoking classifications: non-, occasional, current, and daily smokers.

Sixty-one percent of participants had biological samples obtained within one month of survey administration. Twenty-six of our 382 sub-sample participants (6.8%) had a urine cotinine level above zero. Among these, the median cotinine-creatinine ratio was 207.8 mcg/mg (IQR 119.4–496.1). Median adjusted cotinine was 0.0 mcg/mg (IQR 0–0) for both self-reported non-smokers and occasional smokers in our sample, compared to 172.3 mcg/mg (IQR 0–709.2) for daily smokers. Mean adjusted cotinine was 6.0, 26.0, and 354.4 mcg/mg, for each group, respectively (Fig. 1). The difference in mean adjusted cotinine levels between occasional and non-smoking groups was not significant (*p* = 0.79), while cotinine levels in daily smokers were higher than in both occasional smokers and non-smokers (*p* < 0.001, respectively). In our sub-sample, high adjusted cotinine validated daily smoking (−0.8% difference, *p* = 0.62; Table 2). The sensitivity, specificity, positive predictive value, and negative predictive value of self-reported daily smoking as compared to the gold standard of high cotinine in our sample were: 61.9%, 98.6%, 72.2%, and 97.8%, respectively. The length of time between interview and urine sample did not affect the validity of self-reported daily smoking (Additional file 1: Table S4). The kappa statistic for agreement in classification between the three levels of self-report and the three levels of urine cotinine was 0.40 (95% CI 0.31, 0.45), indicating moderate agreement. Kappa for self-reported daily smokers and creatinine- adjusted cotinine (as dichotomous categories) was 0.65 (95% CI 0.47, 0.83), indicating substantial agreement.

**Table 2:** Comparison of smoking prevalence (%; 95% confidence interval) by ascertainment method and site, CRONICAS Study, Peru, 2010

**Occasional smoking**	**Lima** **(n=195)**	**Tumbes** **(n=187)**	**Tota**l **(n=382)**
Self-Report	10.3 (6.0, 14.5)	7.5 (3.7, 11.3)	8.9 (6.1, 11.8)
Creatinine-Adjusted Cotinine (10–100 mcg/mg)	0.5 (−0.5, 1.5)	2.1 (0.1, 4.2)	1.3 (0.2, 2.4)
Difference by method	**9.7 (5.4, 14.1)**	**5.3 (1.0, 9.7)**	**7.6 (4.5, 10.7)**
**Daily smoking**	**Lima** **(n=195)**	**Tumbes** **(n=187)**	**Total** **(n=382)**
Self-Report	3.1 (0.7, 5.5)	6.4 (2.9, 9.9)	4.7 (2.6, 6.8)
Creatinine-Adjusted Cotinine (≥100 mcg/mg)	2.1 (0.1, 4.0)	9.1 (5.0, 13.2)	5.5 (3.2, 7.8)
Difference by method	1.0 (−2.1, 4.2)	−2.7 (−8.1, 2.7)	−0.8 (−3.9, 2.3)

Bold indicates significance at *p* = 0.05. Differences estimated by t-tests

### Characterization of smokers

Prevalence of reported symptoms, disease diagnoses, group means of continuous biomarkers, and lung testing scores by reported smoking classification for baseline and 40-month follow up can be found in Table 3 and Additional file 1: Table S5. The reported odds ratios and differences in mean represent the occasional and daily smoking groups relative to non-smokers. Additionally, self-reported occasional smokers in our sample had a mean lifetime smoking history of 1.27 pack-years compared to 1.12 pack-years among non-smokers (37% of whom were past smokers at study baseline) (*p* = 0.68). Self-reported daily smokers had a mean smoking history of 7.45 pack-years (*p* < 0.001 vs. occasional and non-smokers), which was comparable to the 9.50 pack years’ history reported by past regular smokers (*p* = 0.26).

**Table 3:** Prevalence and means of chronic disease indicators at baseline and 40 month follow up, CRONICAS Study, multiple sites in Peru, 2010

Outcome (Prevalence)	*N*	Smoking Category	Baseline	Second Follow-Up	Adj. Odds Ratio^a^	*p*-value
Hypertension, %	2583	Non-Smoking	27.2	32.2	---	*---*
		Occasional	19.3	24.0	0.86	0.75
		Daily	31.9	29.7	1.46	0.60
Diabetes, %	2581	Non-Smoking	5.4	7.8	---	---
		Occasional	3.9	6.0	0.82	0.78
		Daily	3.3	8.8	1.31	0.73
Stroke, %	2583	Non-Smoking	0.4	0.5	---	---
		Occasional	0.4	0.4	1.76	0.78
		Daily	1.1	1.1	5.62	0.44
Cardiovascular Disease, %	2583	Non-Smoking	5.0	7.3	---	---
		Occasional	4.3	5.2	1.44	0.58
		Daily	8.8	13.2	10.24	**0.001**
Overweight, %	2541	Non-Smoking	73.6	75.0	---	---
		Occasional	74.6	77.6	1.08	0.90
		Daily	64.8	71.4	0.23	0.09
Obese, %	2541	Non-Smoking	29.3	30.1	---	*---*
		Occasional	24.1	25.9	0.30	0.14
		Daily	20.9	24.2	0.09	**0.035**
Outcome (Mean)	*N*	Smoking Category	Baseline	Second Follow-Up	Adj. Difference in Mean^a^	*p*-value
Systolic Blood Pressure (mmHg)	2543	Non-Smoking	116.8	119.2	---	---
		Occasional	118.2	120.1	2.02	0.06
		Daily	121.2	123.2	0.98	0.54
Body Mass Index (kg/m^2^)	2541	Non-Smoking	27.9	28.1	---	---
		Occasional	27.5	27.9	−0.06	0.86
		Daily	27.0	27.5	−0.74	0.11
HDL (mg/dL)	2411	Non-Smoking	41.2	45.7	---	---
		Occasional	41.3	44.5	1.95	**0.013**
		Daily	40.6	43.6	0.83	0.49
LDL (mg/dL)	2410	Non-Smoking	127.7	120.9	---	---
		Occasional	125.2	116.5	−1.24	0.58
		Daily	125.8	118.7	−1.46	0.67
Total Cholesterol (mg/dL)	2411	Non-Smoking	201.4	198.4	---	---
		Occasional	198.4	192.5	−0.92	0.73
		Daily	196.2	194.4	−2.88	0.47
Triglycerides (mg/dL)	2411	Non-Smoking	163.2	159.0	---	---
		Occasional	159.5	157.5	−9.22	0.15
		Daily	148.9	160.5	−12.52	0.19
HOMA-IR	2410	Non-Smoking	4.6	5.4	---	---
		Occasional	5.3	5.4	0.86	**0.007**
		Daily	4.2	5.3	−0.15	0.75
Framingham Score	2376	Non-Smoking	10.8	11.4	---	---
		Occasional	12.8	11.1	3.26	**<0.001**
		Daily	13.6	13.5	3.57	**<0.001**
Post-Bronchodilator Forced Vital Capacity (L)	2278	Non-Smoking	3.42	3.33	---	---
		Occasional	4.18	4.08	0.05	0.17
		Daily	3.82	3.77	−0.07	0.21
Post-Bronchodilator Forced Expiratory Volume in One Second (L)	2278	Non-Smoking	2.74	2.66	---	---
		Occasional	3.33	3.24	0.02	0.47
		Daily	3.10	2.99	−0.03	0.53

Bold indicates significant results. ^a^Adjusted for age, sex, field site, and wealth index

We modeled the change in lung function between baseline and follow up. Controlling for age, sex, site, and wealth index, daily smokers trended toward having a greater decline in FVC (−1%; 95% CI -2.9, 0.8) and FEV_1_ (−1.3%; 95% CI -3.2, 0.6) over 40 months when compared to non-smokers, whereas the decline in lung function was similar between occasional smokers and non-smokers (−0.2% FVC; 95% CI -1.5, 1.0) and (0% FEV_1_; 95% CI -1.3, 1.3).

Daily smokers trended toward an increase in BMI (+1.10%; 95% CI -0.09, 2.29) compared to occasional smokers (+0.18%; 95% CI -0.61, 0.97) against non-smokers.

Occasional smokers experienced an adjusted IRR for diabetes of 0.94 over follow up compared to non-smokers (95% CI 0.48, 1.84) while the IRR for daily smokers was 1.46 (95% CI 0.63, 3.37). The IRR for hypertension for occasional smokers was 0.99 (95% CI 0.62, 1.56) and 0.73 (95% CI 0.34, 1.57) for daily smokers.

## Discussion

Systematic surveillance of tobacco use is an essential step in the formation of programs to reduce smoking-related mortality, especially for those who joined the WHO’s Framework Convention on Tobacco Control, which obliges signatory nations, including Peru, to implement policy changes to reduce tobacco use [26]. In our primary analysis, based on urine cotinine, we validate a lower prevalence of smoking in low-income peri-urban areas than was reported in urban areas in previous large studies. It appears that Peru is part of a pattern seen in other Latin American countries, with the exception of the Southern Cone, that is characterized by smoking practices favoring occasional over daily smoking and fewer cigarettes consumed. The prevalence of self-reported smoking in our entire sample (*n* = 2978) was 3.3% daily, 8.9% occasional, and 12.2% current smokers. These findings are compatible with GATS data from Mexico [40], Costa Rica [41], and Panama [42]: all of which feature low smoking prevalence and daily smoking equal to or less than half of current smoking. This pattern of reduced tobacco use was previously noted by Tapia-Conyer et al., who found that daily smoking in Mexico decreased from 64% to 52% between 1988 and 1998 while among daily smokers, those who smoked five or fewer cigarettes/day increased from 49% to 74% [43]. Earlier, Samet et al. found that Hispanics in New Mexico tended to consume about half as many cigarettes as Whites, though prevalence of smoking and years smoked were similar [44]. This pattern contrasts with the Southern Cone countries, which have demonstrated higher smoking prevalence and frequency. Both the PLATINO and CARMELA studies report current smoking prevalence of nearly 40% in Chile and Argentina (WHO reports a drop to 29% in Argentina) while figures from GATS show a lower current smoking prevalence of 25% for Uruguay but notes that 80% of current smokers are also daily smokers [3, 45–47]. Menezes et al. note that only 2 of 1626 (0.12%) current smokers in the PLATINO study were *not* daily smokers, contrasting remarkably with our findings in Peru [45].

Our self-reported smoking prevalence mirrors that published by Weygandt et al. but was substantially less than what the CARMELA study reported from Lima [3, 5]. In one comparison, we found 15.5% prevalence of current smoking in peri-urban Lima while CARMELA reported 26.6% prevalence for the 45–65 year age range [3]. For all ages in Lima, we found a current smoking prevalence of 14.8% and for all sites, 12.2%. Additionally, CARMELA reported finding a mean of around 7 cigarettes consumed daily among currently smoking men and around 5 for women [3]. Our study found a mean of 1.8 cigarettes per day for men and 1.1 for women among current smokers in peri-urban Lima. However, it is important to note that these sets of estimates are not directly comparable: generally, CARMELA reported use of all inhaled tobacco in an urban sample of age 25–65 years while we report cigarette use in adults aged ≥35 years living in resource-poor settings [3]. One should also note that the relationship between nicotine intake and cigarettes smoked is not completely linear. Blackford et al. report that, in an international sample, participants tended to titrate smoking to their preferred nicotine level and not number of cigarettes smoked. Subsequently, those who smoked fewer than 20 cigarettes per day (99.5% of our sample) consumed more nicotine per cigarette than those who smoked more [11].

In our secondary analysis, we attempted to characterize the smoking populations of low-income areas using longitudinal data. It is difficult to come to any conclusions about the long-term risk of chronic disease between groups, perhaps because the use of tobacco is so low, even among daily smokers. Only 3% of our entire cohort reported a history of ten or more pack-years. Considering the general guidelines for lung cancer screening (loosely, ≥55 years old and ≥30 pack-years smoking), only 0.75% of our sample qualified [48–51]. Furthermore, there was no difference between mean pack-years for the occasional and non-smoking groups, indicating that the occasionally smoking group had a similar history to the then non-smokers.

Considering this, the relationships between lung function, obesity, and smoking status are of note. Unadjusted means show a greater loss of functional volume (FVC) and ability to exhale (FEV_1_) for daily smokers compared to occasional and non-smokers and the gap was made more apparent by linear regression with age, sex, site, and wealth index, although the difference was not statistically significant. The same pattern was found when observing changes in BMI, with daily smokers experiencing a greater percentage rise in BMI than occasional or non-smokers. That pattern was also exacerbated in linear modeling though, again, it was not significant. No association was found with smoking status and change in systolic or diastolic blood pressures. This indicates that, even with relatively low levels of smoking, daily smokers in this sample may have a worsening risk profile for chronic disease than occasional or non-smokers. However, taking into account the rest of our data, the groups may not be different in a clinical way, which presents opportunities for the Peruvian government to intervene and prevent the public health situation from getting worse.

As an observation, multi-center surveys of smoking prevalence such as PLATINO did not distinguish between current and daily smoking, while those using the GATS protocol, such as CARMELA and our group, did [3, 5, 45]. Under the usual definition of current smoking – a small lifetime history and any reported smoking within a month or more – no value is given to the frequency of cigarette smoking, which, as shown by our sample, may be very low. The definition of active smoking from the PLATINO study would have included all of our self-reported daily smokers and 82% of our low-use occasional smokers, inappropriately characterizing our sample. As Weygandt et al. argue, including those who smoke as infrequently as they do in our sample represents an over-estimation of the actual active smoking prevalence in a population [5].

Strengths of this study include high-quality data collection methods and surveillance of disease outcomes, nested within a well-designed population-based study, and standardized laboratory methods for measuring biomarkers. Limitations of this study include the small sample of daily smokers, which limited statistical power in attempts to characterize the group, and the larger number of participants lost to follow up in the Puno field sites, which may have skewed our health outcomes results. Additionally, the varying time between interview and biological sample question could change our associations but, since people in our sample rarely changed smoking status (our main exposure), we don’t anticipate that the lags had any noticeable effect.

## Conclusions

In summary, we used creatinine-adjusted urine cotinine to validate observed low prevalence of self-reported active smokers and low overall frequency of smoking in low-income areas of Lima and Tumbes, Peru. This places Peru within an emerging pattern of low prevalence and frequency of smoking, favoring occasional smoking, seen in other Latin American countries except for those in the Southern Cone. As Peru is party to WHO’s FCTC as of 2004, it is imperative that national policymakers have access to accurate epidemiological data on tobacco smoking in order to properly implement the MPOWER program.

In investigating longitudinal health outcomes data, we did not observe significant differences between groups of self-reported smokers, perhaps due to low levels of smoking across the population. However, even with low cigarette consumption, patterns can be seen that favor worsening lung function and BMI over time for daily smokers compared to occasional and non-smokers.

As a final observation, we recommend the use of the Global Adult Tobacco Survey protocol – which distinguishes between daily and current smoking – for tobacco use surveillance in future epidemiological studies. This would be especially useful in characterizing regions such as Peru, where the sole use of current smoking as an indicator overestimates the real prevalence of active smoking.

## Additional file

Additional file 1: Table S1. Comparison of cotinine subsample against balance of parent sample. **Table S2**. Comparison of those lost to follow up against those who completed follow up. **Table S3**. Estimates of smoking prevalence with 95% confidence intervals of smoking stratified by age, sex, and site. **Table S4**. Differences in estimates of daily smoking stratified by time between survey and urine sample. **Table S5**. Prevalence and means of chronic disease symptoms at baseline and 40 month follow up. (DOCX 25 kb)

## Abbreviations

BMI: body mass index
EDTA: ethylenediaminetetraacetic acid
FEV_1_: forced expiratory volume in one second
FRS: Framingham risk score
FVC: forced vital capacity
GATS: global adult tobacco survey
HOMA-IR: the homeostasis model assessment of insulin resistance
IQR: interquartile range
LMIC: low and middle income countries
WHO: World health organization
