# Supplementary material for: Low cigarette smoking prevalence in peri-urban Peru: results from a population-based study of tobacco use by self-report and urine cotinine
Source: Tob Induc Dis. 2017 Jul 21;15:32. doi: 10.1186/s12971-017-0137-8 (PMC5521105; doi:10.1186/s12971-017-0137-8)
Supplement: Additional file 1: Table S1. — Comparison of cotinine subsample against balance of parent sample. Table S2. Comparison of those lost to follow up against those who completed follow up. Table S3. Estimates of smoking prevalence with 95% confidence intervals of smoking stratified by age, sex, and site. Table S4. Differences in estimates of daily smoking stratified by time between survey and urine sample. Table S5. Prevalence and means of chronic disease symptoms at baseline and 40 month follow up. (DOCX 25 kb) [file 12971_2017_137_MOESM1_ESM.docx]

**Additional file 1**

**Table 1. Comparison of cotinine subsample against balance of parent sample.**

|  | **Cotinine Subsample** | **Non-subsample** | **p-value** |
| --- | --- | --- | --- |
| *Sociodemographics, n(%) or median (IQR)* | | | |
| n | 382 (19.4) | 1588 (80.6) |  |
| Age | 54.8 (44.7-64.8) | 54.6 (45.1-64.1) | 0.84 |
| Male | 189 (49.5) | 786 (49.5) | 0.99 |
| Education in years |  |  | 0.28 |
| Primary or less | 171 (44.8) | 778 (49.0) |  |
| Secondary | 141 (36.9) | 558 (35.1) |  |
| Higher than secondary | 70 (18.3) | 252 (15.9) |  |
| Wealth Index |  |  | 0.16 |
| Low | 70 (18.3) | 362 (22.8) |  |
| Medium | 153 (40.1) | 607 (38.3) |  |
| High | 159 (41.6) | 616 (38.9) |  |
| Smoking |  |  | 0.91 |
| Non-smoking | 330 (86.4) | 1369 (86.2) |  |
| Occasional | 34 (8.9) | 150 (9.5) |  |
| Daily | 18 (4.7) | 69 (4.4) |  |
|  |  |  |  |
| *Comorbidities and laboratory findings, n (%) or median (IQR)* | | | |
| Diabetes Mellitus | 26 (7.3) | 120 (7.6) | 0.83 |
| HbA1c | 5.8 (5.5-6.0) | 5.8 (5.5-6.1) | 0.53 |
| Hypertension | 78 (21.9) | 365 (23.0) | 0.63 |
| BMI, kg/m^2^ | 28.0 (25.0-31.2) | 27.9 (25.3-31.0) | 0.55 |
| Total cholesterol, mg/dL | 201 (179-229) | 200 (174-226) | 0.38 |
| HOMA-IR | 3.6 (2.1-6.1) | 3.6 (2.1-6.0) | 0.54 |
| Framingham Risk Score |  |  | 0.19 |
| Low | 249 (65.2) | 1063 (67.3) |  |
| Intermediate | 107 (28.0) | 445 (28.2) |  |
| High | 99 (3.9) | 72 (4.6) |  |

**E-Table 2. Comparison of those lost to follow up against those who completed follow up.**

|  | **Lost to follow up** | **Complete follow up** | **p-value** |
| --- | --- | --- | --- |
| *Sociodemographics, n (%) or median (IQR)* | | | |
| N | 394 (13.2) | 2584 (86.8) |  |
| Age | 56.2 (46.0-67.5) | 54.4 (44.9-63.5) | **<0.001** |
| Male | 192 (48.7) | 1271 (49.2) | 0.86 |
| Field Site |  |  | **<0.001** |
| Lima | 98 (24.9) | 906 (35.1) |  |
| Tumbes | 63 (16.0) | 902 (34.9) |  |
| Urban Puno | 112 (28.4) | 391 (15.1) |  |
| Rural Puno | 121 (30.1) | 385 (14.9) |  |
| Education |  |  | **0.01** |
| Primary or less | 204 (51.8) | 1134 (43.9) |  |
| Secondary | 114 (28.9) | 882 (34.1) |  |
| Higher than Secondary | 76 (19.3) | 568 (22.0) |  |
| Wealth Index |  |  | **<0.001** |
| Low | 157 (40.0) | 751 (29.1) |  |
| Middle | 126 (32.1) | 898 (34.8) |  |
| High | 110 (28.0) | 933 (36.1) |  |
| Smoking Status |  |  | 0.25 |
| Non-Smoker | 354 (89.9) | 2260 (87.5) |  |
| Occasional | 32 (8.1) | 223 (9.0) |  |
| Daily | 8 (2.0) | 91 (3.5) |  |
|  | | | |
| *Comorbidities and laboratory findings, n (%) or median (IQR)* | | | |
| Hypertension | 111 (28.1) | 689 (26.7) | 0.55 |
| Diabetes | 26 (6.6) | 134 (5.2) | 0.25 |
| Stroke | 5 (1.3) | 12 (0.5) | **0.05** |
| CVD | 14 (3.5) | 132 (5.1) | 0.18 |
| Overweight, BMI ≥ BM | 257 (65.1) | 1891 (73.2) | **0.001** |
| Obese, BMI ≥ 30 | 103 (26.1) | 734 (28.4) | 0.36 |
| BMI, kg/m^2^ | 26.7 (23.5-30.1) | 27.5 (24.7-30.4) | **0.002** |
| Systolic BP, mmHg | 115.5 (106-129) | 114 (104.5-126.5) | **0.01** |
| Framingham Score | 13 (9-15) | 12 (8-14) | **0.05** |
| HDL Cholesterol, mg/dL | 40.1 (35-48) | 39.5 (33-47.9) | 0.32 |
| LDL Cholesterol, mg/dL | 120.7 (100.1-144.3) | 124.8 (103.3-148.4) | 0.08 |
| Total Cholesterol, mg/dL | 193 (170-219.5) | 198 (173-225) | **0.05** |
| Triglycerides, mg/dL | 133 (94-185.5) | 137 (99-193) | 0.18 |
| Post-BD FVC, L | 3.38 (2.68-4.37) | 3.37 (2.73-4.16) | 0.40 |
| Post-BD FEV_1_, L | 2.74 (2.13-3.51) | 2.71 (2.21-3.34) | 0.83 |

**E-Table 3. Estimates of smoking prevalence with 95% confidence intervals of smoking stratified by age, sex, and site.**

|  | **Lima**  **(n = 1004)** | | **Tumbes**  **(n = 965)** | | **Urban Puno**  **(n = 503)** | | **Rural Puno**  **(n = 506)** | | **Overall**  **(n = 2978)** | |
| --- | --- | --- | --- | --- | --- | --- | --- | --- | --- | --- |
|  | Daily | Current | Daily | Current | Daily | Current | Daily | Current | Daily | Current |
| Overall |  |  |  |  |  |  |  |  |  |  |
| All | 3.3 (2.3-4.6) | 14.8 (12.8-17.2) | 5.6 (4.3-7.3) | 12.6 (10.7-14.9) | 2.2 (1.2-3.9) | 11.1 (8.7-14.2) | 0.2 (0.0-1.4) | 7.3 (5.3-9.9) | 3.3 (2.7-4.0) | 12.2 (11.1-13.4) |
| Men | 5.1 (3.5-7.4) | 24.8 (21.2-28.8) | 9.7 (7.4-12.7) | 21.9 (18.5-26.9) | 2.4 (1.1-5.3) | 16.9 (12.7-22.1) | 0.4 (0.0-2.9) | 13.4 (9.6-18.3) | 5.4 (4.4-6.7) | 20.6 (18.6-22.8) |
| Women | 1.6 (0.8-3.1) | 5.3 (3.6-7.6) | 1.5 (0.7-3.0) | 3.3 (2.0-5.4) | 2.0 (0.8-4.7) | 5.5 (3.3-9.1) | n/a | 1.9 (0.8-4.5) | 1.3 (0.9-2.0) | 4.1 (3.2-5.2) |
| <45 |  |  |  |  |  |  |  |  |  |  |
| A | 2.9 (1.4-6.0) | 20.0 (15.4-25.6) | 4.4 (2.4-7.8) | 16.4 (12.3-21.5) | 3.2 (1.2-8.2) | 15.1 (9.8-22.5) | 0.8 (0.1-5.7) | 9.9 (5.7-16.7) | 3.1 (2.1-4.7) | 16.3 (13.8-19.1) |
| M | 5.0 (2.2-10.6) | 36.4 (28.3-45.3) | 7.3 (3.8-13.5) | 30.1 (22.6-38.9) | 3.2 (0.8-12.2) | 25.8 (16.4-38.2) | 1.8 (0.2-12.0) | 20.0 (11.4-32.8) | 5.0 (3.2-7.8) | 29.9 (25.4-34.9) |
| W | 0.8 (0.1-5.8) | 3.4 (1.3-8.7) | 1.6 (0.4-6.1) | 3.1 (1.2-8.1) | 3.1 (0.8-11.8) | 4.7 (1.5-13.7) | n/a | 1.5 (0.2-10.2) | 1.3 (0.6-3.2) | 3.2 (1.8-5.5) |
| 45-54 |  |  |  |  |  |  |  |  |  |  |
| A | 4.0 (2.2-7.1) | 19.0 (14.8-24.2) | 9.3 (6.2-13.8) | 18.6 (14.2-24.1) | 2.3 (0.8-7.1) | 18.0 (12.2-25.6) | n/a | 6.7 (3.5-12.4) | 4.7 (3.4-6.4) | 16.6 (14.1-19.4) |
| M | 7.1 (3.7-13.1) | 31.5 (24.0-40.1) | 15.8 (10.3-23.5) | 31.7 (23.9-40.6) | 3.1 (0.8-11.6) | 26.2 (16.8-38.2) | n/a | 12.1 (5.8-23.4) | 8.1 (5.7-11.4) | 27.6 (23.2-32.4) |
| W | 1.4 (0.3-5.3) | 8.2 (4.7-13.9) | 2.6 (0.8-7.8) | 5.2 (2.3-11.1) | 1.6 (0.2-10.6) | 9.5 (4.3-19.8) | n/a | 2.6 (0.7-10.0) | 1.5 (0.7-3.3) | 6.5 (4.4-9.4) |
| 55-64 |  |  |  |  |  |  |  |  |  |  |
| A | 3.9 (2.1-7.1) | 11.7 (8.3-16.3) | 6.1 (3.7-9.9) | 11.8 (8.3-16.5) | 3.1 (1.2-8.1) | 7.8 (4.2-14.0) | n/a | 5.9 (3.0-11.4) | 3.8 (2.6-5.4) | 10.1 (8.1-12.4) |
| M | 4.8 (2.2-10.3) | 17.6 (11.9-25.3) | 11.9 (7.1-19.1) | 20.3 (14.0-28.6) | 3.2 (0.8-12.0) | 11.1 (5.3-21.7) | n/a | 12.9 (6.5-23.9) | 6.0 (4.0-8.9) | 16.6 (13.1-20.7) |
| W | 3.1 (1.1-7.9) | 6.1 (3.1-11.8) | 0.8 (0.1-5.4) | 3.9 (1.6-9.1) | 3.1 (0.8-11.6) | 4.6 (1.5-13.5) | n/a | n/a | 1.8 (0.8-3.7) | 4.0 (2.5-6.5) |
| 65+ |  |  |  |  |  |  |  |  |  |  |
| A | 2.1 (0.9-5.0) | 8.1 (5.2-12.3) | 2.6 (1.2-5.6) | 3.4 (1.7-6.7) | n/a | 3.3 (1.2-8.5) | n/a | 7.0 (3.5-13.5) | 1.6 (0.9-2.8) | 5.5 (4.1-7.5) |
| M | 3.4 (1.3-8.7) | 13.4 (8.4-20.4) | 4.1 (1.7-9.5) | 5.7 (2.7-11.6) | n/a | 3.4 (0.8-12.7) | n/a | 9.4 (4.2-19.5) | 2.5 (1.3-4.7) | 8.5 (6.0-11.9) |
| W | 0.9 (0.1-5.9) | 2.6 (0.8-7.8) | 0.9 (0.1-6.1) | 1.0 (0.1-6.1) | n/a | 3.2 (0.8-12.2) | n/a | 4.1 (1.0-15.1) | 0.6 (0.1-2.3) | 2.4 (1.2-4.7) |

**E-Table 4. Differences in estimates of daily smoking stratified by time between survey and urine sample.**

| **Difference in Collection Date** | **Self-Reported Daily Smoking** | **Adjusted Urine Cotinine >100 mg/mg** | **Difference in Estimates** |
| --- | --- | --- | --- |
| Within 1 week | 2 (1.42%) | 2 (1.42%) | 0.00% |
| 1 week - 1 month | 6 (6.59%) | 7 (7.69%) | 1.10% |
| > 1 month | 10 (6.67%) | 12 (8.00%) | 1.33% |

**E-Table 5. Prevalence and means of chronic disease symptoms at baseline and 40 month follow up.**

| **Outcome (Prevalence)** | **N** | **Smoking Category** | **First Sweep** | **Third Sweep** | **Odds Ratio** | **p-value** |
| --- | --- | --- | --- | --- | --- | --- |
| Cough | 2580 | Non-Smoking | 4.7 | 13.2 | --- | --- |
|  |  | Occasional | 2.1 | 14.2 | 1.13 | 0.57 |
|  |  | Daily | 1.1 | 5.5 | 0.57 | 0.23 |
| Phlegm | 2581 | Non-Smoking | 6.0 | 13.0 | --- | --- |
|  |  | Occasional | 3.9 | 13.7 | 1.08 | 0.72 |
|  |  | Daily | 0.0 | 4.4 | 0.36 | *0.10* |
| Wheeze | 2582 | Non-Smoking | 4.9 | 11.7 | --- | *---* |
|  |  | Occasional | 3.4 | 12.0 | 1.08 | 0.76 |
|  |  | Daily | 0.0 | 4.4 | 0.36 | *0.08* |
